# Verifying the Stability of Selected Genes for Normalization in Q PCR Experiments of *Spodoptera frugiperda* Cells during AcMNPV Infection

**DOI:** 10.1371/journal.pone.0108516

**Published:** 2014-10-14

**Authors:** Tamer Z. Salem, Walaa R. Allam, Suzanne M. Thiem

**Affiliations:** 1 Department of Biomedical Sciences, University of Science and Technology, Zewail City of Science and Technology, Giza, Egypt; 2 Department of Microbial Molecular Biology, AGERI, Agricultural Research Center, Giza, Egypt; 3 Department of Entomology, Michigan State University, East Lansing, Michigan, United States of America; 4 Department of Microbiology and Molecular Genetics, Michigan State University, East Lansing, Michigan, United States of America; Institute of Vegetables and Flowers, Chinese Academy of Agricultural Science, China

## Abstract

It is challenging to find genes with stable transcripts for use as reference genes for quantitative realtime polymerase chain reaction (qRT-PCR) during viral infection. *Autographa californica* nucleopolyhedrovirus (AcMNPV) is known to globally shut off host gene transcription in Sf21 cells and to modify their cytoskeletons. In this study, seven host genes were selected for validation as references for gene expression experiments using qRT-PCR. Two of them, ecdysoneless (ECD) and myosin showed stable RNA levels in our previous microarray study at 6, 12, and 24 hpi for both genes and 48 hpi for ECD. The others, actin, tubulin, glyceraldehyde 3-phosphate dehydrogenase (GAPDH), and 28S ribosome (28S), are commonly employed as reference genes for qRT-PCR. Ribosomal protein L35 (L35) gene was selected to test if ribosomal protein genes show stable RNA transcript levels similar to 28S and 18S rRNA and to validate the microarray data. In addition to 28S, previously known to have stable transcript levels, qRT-PCR showed that ECD transcript levels remained constant throughout the time course of AcMNPV infection. Transcripts of cytoskeleton genes such as actin, tubulin, and myosin declined dramatically as the infection progressed. GAPDH and L35 transcripts also declined over time. These results indicate that ECD is a reliable reference gene for qRT-PCR experiments during AcMNPV infection of *Spodoptera frugiperda* cells. Although 28S could be used as a reference gene for these experiments, it is less useful than ECD because of its abundance, which might make it difficult to establish an accurate baseline value for data analysis.

## Introduction

Quantitative real-time PCR (qRT-PCR) is considered the gold standard for accurate measurements of gene expression. However, qRT-PCR is extremely sensitive necessitating careful selection of an internal control gene with stable transcripts. Genes involved in basic cell maintenance processes are expected to maintain stable expression levels among different tissues and conditions; these “housekeeping” genes, have long been used as control or “normalization” genes [1]. However, transcript levels of many of these genes varied in response to tissue, cell lines or experimental conditions. For example transcripts of the classical reference genes, beta actin (β-actin) and glyceraldehyde 3-phosphate dehydrogenase (GAPDH), varied greatly during bacterial and viral infection. Actin and GAPDH RNA levels showed >20–35 fold change in human peripheral blood mononuclear cells (PBMCs) stimulated with tuberculosis antigens [2]. β-actin transcript levels fluctuated in different human cell lines infected with coronavirus, yellow fever virus, human herpesvirus-6, cytomegalovirus, or camel poxvirus, indicating that β-actin is not a suitable reference gene for these virus-infected cells [3]. Actin and GAPDH transcript levels also varied widely under conditions such as hypoxia [4], presence of specific proteins [5] or diseases, such as asthmatic airways [6].

Because variations in reference-gene RNA levels between different samples will compromise the reliability of qRT-PCR analysis, it was suggested that more than one stably expressed reference gene should be used to avoid misinterpreting gene expression profiles [1]. Moreover it was found that no gene is capable of maintaining constant levels of expression under all conditions; making it necessary to evaluate reference genes for each new experimental condition [7]. This is particularly important when studying host gene expression in virus-infected cells because viruses manipulate cellular protein expression and interfere with the basic host cell pathways such as cell cycle, metabolism, DNA replication and transcription to favor their own replication [8], [9], [10]. In the case of baculovirus-infected cells, host gene expression showed global down-regulation by 12–18 hour post infection (hpi) in *Bombyx mori* nucleopolyhedrovirus-infected *B. mori* BmN-4 cells [11]. In *Autographa californica* multiple nucleopolyhedrovirus (AcMNPV)-infected *Spodoptera frugiperda* (Sf21) cells, actin, histone, and heat shock 70 mRNAs were greatly reduced between 12 and 18 hpi and eukaryotic translation initiation factor eIF4E mRNAs decreased between 12 and 24 hpi [12], [13]. We previously reported that AcMNPV infection of Sf21 cells caused a substantial decline in transcript levels of nearly all host genes represented on a microarray (approximately 11,000 ESTs) from 12 to 24 hpi [10]. In addition, differential display analysis showed a decrease in host mRNA levels between 12–24 hpi in AcMNPV-infected Sf9 cells [13], [14], [15]. In this study, we evaluated seven candidate reference genes, including four of the commonly used housekeeping genes (actin, GADPH, tubulin and 28S), for normalizing qRT-PCR in AcMNPV-infected Sf21 cells.

## Materials and Methods

### Cell line and virus

Sf21 cells [16] were maintained at 27°C in TC-100 media (Sigma) supplemented with 10% fetal bovine serum (Atlanta Biologicals). Wild-type AcMNPV strain L1 virus [17] used in this study and titrated by plaque assay followed standard procedures [18] to calculate the plaque forming unit (pfu)/ml. Plaque assays were performed in duplicate on three independent samples. All infections were carried out at multiplicity of infection (moi) of 10.

### Total RNA extraction

A total of 2×10^6^ Sf21 cells were infected with wild-type AcMNPV. TC-100 medium was used for the mock infection. Infected cells were collected at 6, 12, 24 and 48 hpi and mock-infected cells were collected at 12 h after the experiment began. Total RNA was purified by an RNAeasy Mini Kit (Qiagen) according to the manufacturer instructions. The RNA quality was assessed by Agilent 2100 Bioanalyzer using Agilent RNA 6000 Nano LabChip kit. Forty eight hpi was the last time point tested because it is well documented that host gene transcripts decline before 48 hpi during AcMNPV infection [10]. In addition, after that time point, cells will start to die and the RNA concentration will not be representative of the starting number of cells.

### Qualitative real time polymerase chain reaction (qRT-PCR)

The qRT-PCR method was used to evaluate the potential of seven genes (Table 1) as internal reference genes in qRT-PCR assays during AcMNPV infection. A total of 14 forward and reverse primers (Table 1) were designed using Primer Express Software (Applied Biosystems). Five hundred ng of total RNA from each sample was used as a template to synthesize cDNA according to the iScript cDNA synthesis kit manufacturer's instructions (Bio-Rad). The qPCR was conducted using Power SYBR Green PCR Master Mix on 7900HT Fast Real-Time PCR System (Applied Biosystems). Four biological samples for each time point of infection and mock infection were used in the qRT-PCR, and each was conducted in duplicate. The relative transcript level of each gene was calculated according to the 2^−Ct^, for unnormalized genes, and the 2^−ΔΔCt^ method, for the genes normalized to 28S [19]. The relative transcript levels in the mock-infection sample was considered to be 1 and the relative transcript levels of each of the rest of the samples was calculated accordingly. A Student's t-test was performed on the data.

**Table 1:** A list of primers used in this study.

Gene	Primer name	Sequence
**myosin**	myosin F	GGATGACGACGGCATGATC
	myosin R	GCCTCTACGCCATCACCTTCT
**28S**	q28s F	CTGGCTTGATCCAGATGTTCAG
	q28s R	GGATCGATAGGCCGTGCTT
**Tubulin**	Tub F	GGGCATGGACGAGATGGA
	Tub R	GGACACCAGGTCGTTCATGTT
**Ecdysoneless**	ECD F	GCCGTATCCAAAGCATGACA
	ECD R	TGGTGACGGCCAAAGGAA
**L35**	L35 F	GCAACCAGCACGAGAACACA
	L35 R	GCGTCTTCACGGCTCTTTG
**Actin**	Actin F	TTGCCCTGAAGCCCTCTTC
	Actin R	CTCGTGGATACCGCAAGATTC
**GAPDH**	GAPDH F	CGTGGGAGCAGGTTTCGT
	GABDH R	ACGGCCGCTTTAGTGTTGTC

## Results

Studies investigating the selection of reference genes for normalizing qRT-PCR experiments in AcMNPV-infected Sf21 cells are limited. To evaluate the potential of seven candidate reference genes, RNA levels of these genes were analyzed by qRT-PCR in two technical replicates of each of four biological replicates in mock- and AcMNPV-infected Sf21 cells over time.

### Expression Profiles of Candidate Reference Genes

**Figure 1:**
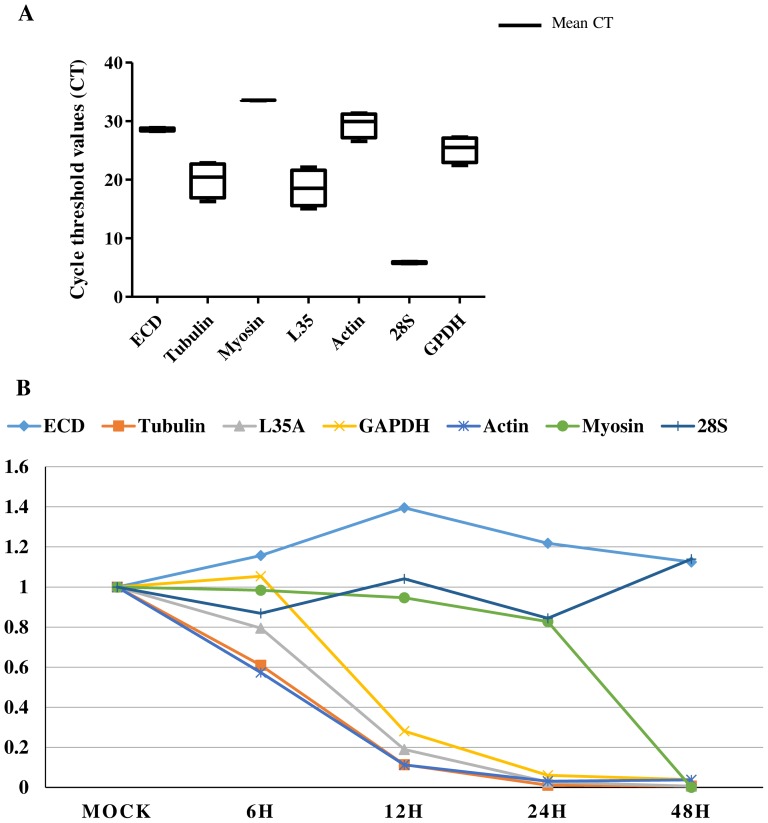
Expression levels of the candidate reference genes. (A) Expression levels displayed as average cycle threshold (Ct) values ±SD of the genes selected for this study, represented in a box and whisker diagram. Whiskers represent the maximum and minimum values. The genes were ordered from the least (higher Ct, to the left) to the most abundantly expressed (lower Ct, to the right) the black line represents the mean of duplicate samples, and the bars indicate the standard deviation of the mean. (B) Fluctuation of relative expression in of the evaluated reference genes AcMNPV-infected Sf21 cells, at 6, 12, 24 and 48 hpi based on the 2^−Ct^ methods.

RNA levels were determined as the number of cycles needed for the amplification to reach a fixed threshold in the exponential phase of the PCR reaction. The overall cycle threshold values (Ct) were compared for the different genes. Each of the seven candidate reference genes displayed a narrow range of mean Ct values across all experimental samples (Fig. 1A). However they exhibited a wide range of RNA levels (Table 2). The fluorescence peak after 5.54 cycles showed that 28S was the most abundant RNA, whereas myosin was the least abundant RNA with a Ct value of 33.5.

**Table 2:** Comparison of average Ct±SD during 48 hpi of AcMNPV, of the seven selected reference genes.

	ECD	Tubulin	Myosin	L35	Actin	28S	GAPDH
**Mock**	28.7±0.60	15.5±0.17	33.4±0.50	14.7±0.21	25.8±0.57	5.54±0.19	22.5±0.21
**6 h**	28.5±0.33	16.2±0.14	33.5±0.57	15.0±0.26	26.6±0.27	5.76±0.28	22.4±0.13
**12 h**	28.2±0.47	18.9±0.75	33.6±0.56	17.2±0.42	29.1±0.83	5.70±0.25	24.4±0.39
**24 h**	28.6±0.74	22.0±0.39	33.7±0.25	19.9±0.41	31.3±1.27	5.95±0.30	26.6±0.35
**48 h**	28.9±1.12	22.9±0.74	undetected	22.1±0.69	30.8±1.12	6.00±1.24	27.2±0.57
**Avg**	28.5±0.24	20.0±3.04	33.5±16.6	18.5±3.12	29.4±2.16	5.86±0.15	25.1±2.20

The highest RNA levels (lowest Cts) for all tested genes were at 6 hpi. Ct results showed a sharp decrease in the transcript levels of tubulin, actin, GAPDH, L35 by 12–24 hpi. In contrast, Ct values of ECD and 28S RNA levels were relatively stable across all examined infection time points (Fig. 1B). Whereas myosin maintained a stable, although high, Ct during first 24 hpi, it was undetectable at 48 hpi (Table 2). For the genes with the least variable RNA levels (28S and ECD), Ct ranges were between 5.54–6.00 and 28.5–28.9, respectively. However, Ct values of the other genes spanned a greater range over the course of infection; tubulin (16.27–22.9), beta actin (26.6–30.8), GAPDH (22.4–28.9), and L35 (15.0–22.1). The myosin gene Ct values ranged between 33.5 and undetectable (lowest, median, and highest Cts of each gene during 48 hpi are shown in Fig. 1).

### Variability of transcript levels

#### A. Transcript levels at different times post infection

To evaluate the suitability of each of the selected genes as reference genes for qRT-PCR experiments in AcMNPV-infected Sf21 cells their mean Ct values and SDs were compared (Table 2). A low average Ct value with low standard deviation (SD) value indicates a higher transcript copy number with a little variation between infection time points, which are characteristics of a reliable reference gene during AcMNPV infection. In contrast, a high SD for the average Ct value of the combination of all of the infection time points indicates that the host gene transcript levels vary over time in the virus-infected cells, making it a poor choice as a reference gene. In addition to being the most abundant RNA (lowest Ct value), 28S rRNA levels fluctuated the least over the course of infection. According to the calculated SD of the mean Ct values, the 28S had the smallest SD value (0.15) followed by ECD, myosin, actin, GAPDH, tubulin and L35 (Table 2).

**Figure 2:**
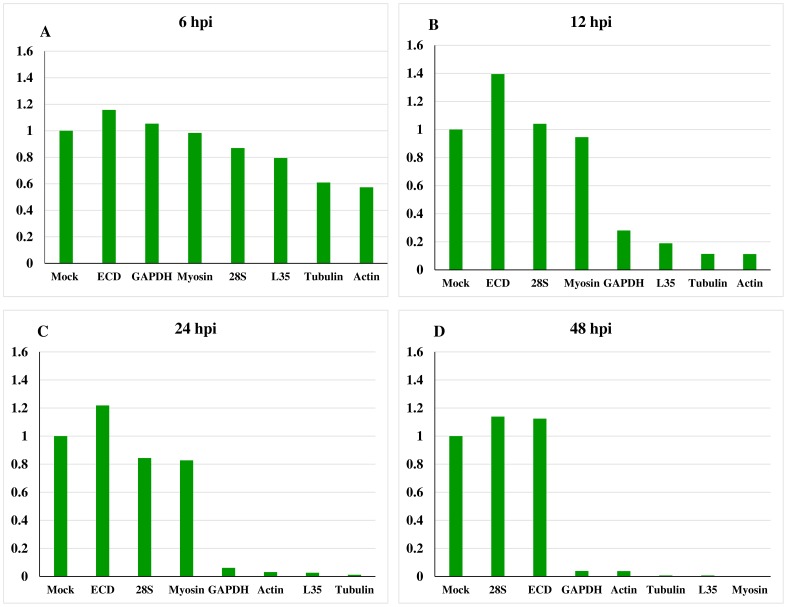
Effect of AcMNPV infection on the expression profile of the selected host genes. Sf21 cells infected with AcMNPV at moi of 10 were harvested at 6, 12, 24, 48 hpi.

The ECD RNA levels remained relatively constant throughout the infection, with the highest and lowest relative expression detected at 12 and 48 hpi, respectively. However, the ECD RNA levels were higher in infected cells compared to the mock infection at all-time points. The RNA levels of all the seven genes were compared to each other in each of the infection time point from 6–48 hpi (Fig. 2A–D).

#### B. Variation in transcript levels post normalization with 28S

**Figure 3:**
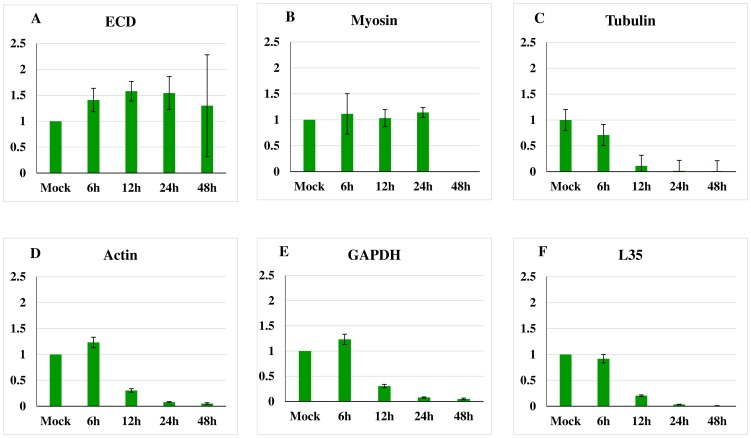
Regulation of gene expression by qRT-PCR. RNA was extracted from mock-infected *Sf*21 cells harvested at 12 h.p.i. and from infected cells harvested at 6, 12, 24, or 48 h.p.i. Primers are listed in Table 1. Each value represents two technical replicates of each of four biological replicates. Standard deviation is indicated by bars. 28S gene was used as internal control for normalization and to calculate the relative expression based on 2^−ΔΔCt^ method [19].

To further evaluate the candidate reference genes during viral infection their transcript levels were normalized to 28S rRNA (known to be stable during AcMNPV infection). The change in RNA levels was recorded using the 2^−ΔΔCt^ method. ECD RNA levels was confirmed up to 48 hpi (Figure 3A), as was the reduction of the RNA levels and inconsistency of tubulin, actin, GAPDH, L35 and myosin RNA levels during AcMNPV infection (Figure 3 B, C, D, E, F, respectively).

## Discussion

This study was conducted to identify host transcripts with minimal fluctuation in abundance as reference genes for qRT-PCR studies of AcMNPV-infected *S. frugiperda* cells. Because they are abundant and maintain stable levels during infection 28S and 18S rRNAs were used to normalize qRT-PCR in various studies on virus-infected insect, vertebrate, and plant cells, [20], [21], [22], [23], [24]. Previously 28S rRNA was shown to be a promising reference gene to normalize qRT-PCR data in baculovirus-infected cells and for infections by other insect DNA viruses such as *Musca domestica* salivary gland hypertrophy virus [10], [20]. In this study, 28S rRNA showed the lowest Ct score and least variation in RNA levels during viral infection, confirming the value of 28S rRNA as a reference for normalizing qRT-PCR gene expression data for AcMNPV-infected *S. frugiperda* cells. However 28S rRNA is not optimal as a reference gene because it is so much more abundant than potential target mRNA transcripts. This makes it difficult to correctly subtract the baseline value in real-time RT-PCR data analysis, which may yield inaccurate conclusions [25].

With the exception of 28S rRNA, none of the other host genes commonly used for normalizing qRT-PCR, actin, tubulin, and GAPDH, which were evaluated in this study, were suitable as reference genes in AcMNPV-infected Sf21 cells. In fact, the greatest variation in mRNA levels in this study was for β-actin, one of the most commonly used reference genes. This result is consistent with a previous study in which northern blot analysis showed a substantial decrease in actin mRNA between 12 and 18 hpi in AcMNPV-infected *S. frugiperda* cells [12]. The microarray data showed that actin was down-regulated by 1.2, 16, and 75 fold at 6, 12, and 24 hpi, respectively (unpublished data). These results indicate that β-actin is a poor choice as a qRT-PCR reference gene in AcMNPV infected *S. frugiperda* cells. We also observed significant drops in both tubulin and GAPDH transcript levels by 12 hpi, indicating that neither is suitable for use as a reference gene in AcMNPV-infected Sf21 cells. However, each of these genes may be useful for normalizing qRT-PCR experiments in other situations involving infected insects or insect cells. For example, tubulin may be suitable for normalizing data in an AcMNPV-infected *Lymantria dispar* cell line. Although neither LdFB but not Ld652Y cells are fully permissive for AcMNPV, tubulin transcript levels showed very different patterns [26]. There were no consistent changes (decreases or increases) in tubulin transcript levels observed in AcMNPV-infected LdFB cells over a 72-h measurement period, suggesting tubulin transcripts could be used for normalization of qRT-PCR data from these infections. In contrast, in AcMNPV-infected Ld652Y cells tubulin transcript levels were greatly reduced after 24 hpi indicating it would be a poor choice for normalizing qRT-PCR data in these infections. GAPDH plays a role in energy metabolism and is one of the most frequently used reference genes [27]. However, in this study GAPDH transcript levels declined significantly by 12 hpi in AcMNPV-infected Sf21 cells. But in bacteria-challenged honeybees, GAPDH transcript levels remained constant [28]. Thus while GAPDH is not suitable for normalizing qRT-PCR data from AcMNPV-infected *S. frugiperda* cells, it should not be rejected for other experimental situations.

One of the host genes identified as a potential normalization gene based on our microarray data proved to be the most suitable for reference gene for experiments on AcMNPV-infected Sf21 cells. ECD transcript levels were the least variable of all of the genes evaluated by the qRT-PCR over the course of AcMNPV infection in Sf21 cells in this study, as they were in our previous microarray study [10]. How ECD transcript levels remain stable during AcMNPV-infection, while most host gene transcript levels decline precipitously is unknown. Myosin, another gene identified as stable in the microarray study, maintained relatively constant transcript levels through 24 hpi before declining by 48 hpi in this qRT-PCR analysis (Fig. 3). This suggests it could potentially be used as a reference gene for experimental data collected by 24 hpi or earlier. However, the Ct score for myosin of 33.5 indicates myosin transcripts are relatively low abundance to a level that makes it difficult to be compared with if used as a reference gene. It is well documented that due to experimental limitations of the PCR reaction, its DNA product is not amplified exponentially and the DNA synthesis will be saturated after 30 to 35 cycles [29]. Ribosomal protein L35, exhibited an expression pattern that was similar to tubulin, actin, and GAPDH in qRT-PCR analysis, declining rapidly after 6 hpi (Fig. 3), inconsistent RNA levels were also observed on microarrays (unpublished data) indicating that L35 is not a good choice for normalizing qRT-PCR in AcMNPV-infected Sf21 cells.

In conclusion, because the majority of host genes are dramatically down-regulated over the course of infection in AcMNPV-infected Sf21 cells [10], [14], choices of reference genes as reliable normalizers for qRT-PCR analyses are limited. We found that both 28S rRNA and ECD transcripts remained relatively constant throughout the course of infection, indicating they can serve as reliable normalizers for qRT-PCR analyses in AcMNPV-infected cells through 48 hpi. However, ECD could be a better choice than 28S RNA for comparing transcripts with mid-range numbers during AcMNPV infection. Myosin is also a potential reference gene as its transcript levels were stable up until 24 hpi. However, we recommend that genes with Cts >30 cycles, as myosin, should not be further considered as a reference gene in AcMNPV infected Sf21 cells since their transcripts are close to the noise that results in with the non-template samples [29]. Surprisingly, actin was the most variable gene in our study and should be avoided in gene expression studies involving AcMNPV-infected *S. frugiperda* cells.
